# Herbicide dose-response thresholds in sands to assess the risk of non-target damage to winter grain crops

**DOI:** 10.1371/journal.pone.0330225

**Published:** 2025-08-21

**Authors:** Win Win Pyone, Richard W. Bell, Michael T. Rose, Gavan S. McGrath

**Affiliations:** 1 SoilsWest, Center for Sustainable Farming Systems, Food Futures Institute, Murdoch University, Australia; 2 Cooperative Research Centre for High Performance Soils, Callaghan, New South Wales, Australia; 3 Biodiversity and Conservation Science, Department of Biodiversity, Conservation and Attractions, Kensington, Washington, Australia; 4 Faculty of Science and Engineering, Southern Cross University, Lismore New South Wales, Australia; La Trobe University - Bundoora Campus: La Trobe University, AUSTRALIA

## Abstract

Herbicide residues in soil from previous crops or from pre-emergent treatments can have unintended toxicity on the next crop. Despite this there is limited published information on toxicity thresholds for many crops or herbicides. This study aimed to quantify shoot and root responses of six common winter grains crops (canola, chickpea, fieldpea, lentil, lupin and wheat) to increasing concentration of four common herbicides (clopyralid, pyroxasulfone, propyzamide and trifluralin) in soil. Lentil emergence was highly sensitive to clopyralid (29 μg kg^-1^ for a 50% reduction, ED_50_) while wheat emergence was sensitive to propyzamide and trifluralin, with complete inhibition at 100 μg kg^-1^ and 375 μg kg^-1^, respectively. Shoot and root parameters of the legumes, except lupin, were significantly reduced by clopyralid, with ED_50_ values ranging between 3−27 μg kg^-1^. Canola was sensitive to pyroxasulfone, with shoot and root biomass ED_50_ at 21 and 8 μg kg^-1^, respectively. Pyroxasulfone also severely reduced root length of all tested crops (ED_50_ values 6−53 μg kg^-1^). Root and shoot growth in wheat was most susceptible to propyzamide followed by trifluralin. This study found that one or more herbicides had the potential to cause significant phytotoxic effects in all crops at concentrations below recommended application rates and below those detected in a recent field survey of pre-sowing herbicide residues in field soils around Australia. These results suggest the risk of early crop damage residual herbicides in very light-textured soils. More effort is now required to determine potential effects on different soil types and crop yields, to enable better spatial and economic risk assessment.

## Introduction

The global use of herbicides in agriculture grew by 15.5% (by mass applied) between 2011 and 2021 [1], driven by the shift toward minimum tillage practices and reduced mechanical weed control [2,3]. In Australia the application of herbicides for controlling weeds, instead of practicing tillage, retained more water in soil profiles and increased grain yields by 15–25% [4]. However, while herbicides are designed to dissipate to minimise residual phytotoxicity to subsequent crops, herbicides can persist longer under certain conditions [5], causing bioactive residues that harm non-target crops [6–8]. For example, imazapyr and imazamox in Argentina reduced barley yields by 45%, and can significantly harm rotational crops like barley, oat, and wheat [9], while mesotrione soil residues in Canada caused up to 100% yield loss in sugar beet [10]. Soil residual herbicides persisting for 12–24 months can impair the growth of subsequent crops (e.g., barley, chickpeas, field peas, lentils, lupins, wheat), posing challenges for sustainable agricultural practices [11]. To minimize the risk of phytotoxicity from residual herbicides, farmers are guided by label-recommended plant-back periods, which are based on field research of chemical carryover in the soil and effect on subsequent crops [12]. However, fluctuating environmental conditions and the variable nature of herbicide persistence in soil makes it difficult for product labels to fully mitigate crop damage under all possible scenarios [13,14]. Carryover of herbicides in soil is influenced by environmental, edaphic and management factors. Soil physical and chemical properties and microbial activity can influence herbicide sorption, mobility, and degradation which determine the herbicide persistence [15–18]. Physicochemical properties of each herbicide, including vapor pressure, water solubility, ionization constant and chemical structure also influence persistence [17]. Soil-active herbicides vary widely in their persistence, with half-lives ranging from a few days to over a year. For instance, the estimated soil half-lives of fomesafen, imazethapyr, clopyralid, and mesotrione are approximately 100, 60–90, 40, and 5–15 days, respectively [19]. Even herbicides with relatively short soil half-lives, like saflufenacil and topramezone, can harm sensitive crops [20], underscoring the need for crop- and environment-specific plant-back intervals beyond general label recommendations. Hence, predicting herbicide carryover is challenging because of multiple factors influencing the dissipation of bio-active forms in soil.

In Australia, herbicides from groups 3, 4 and 15 have been identified as priority herbicides for the study of potential adverse effects on crop production because of their widespread use [21] and their persistence over relatively long time periods [22]. Herbicides from these groups were evaluated in this study and specific attributes of these chemicals are briefly discussed next, illustrating their persistence.

Clopyralid (Group 4- Pyridines) is a synthetic auxin type herbicide [23,24] and can disturb cell respiration and plant growth. This herbicide has a wide range in an half-lives under field conditions, ranging from 15 to >280 days [23]. Pyroxasulfone is a pyrazole-based (Group 15- Isoxazolines) preplant, pre-emergence, and post-emergence herbicide [19] that disturbs shoot elongation of susceptible crop seedlings through inhibiting the biosynthesis of very-long-chain fatty acids [25]. It is one of the most common soil-applied herbicides in Western Australia in predominantly zero-tillage systems [26–28]. The half-life of pyroxasulfone ranges from 47 to 134 days and varies by soil type [29], but in dry years it can persist with a half-life greater than 70 days [30].

Propyzamide is a selective systemic herbicide in the benzamide group (Group 3- Benzamides) and can be absorbed by the roots of the plants. It is resistant to chemical degradation, thus can persist longer in the soil, degrading mostly by photolysis with a half-life of 249 days. However, it also persist longer under anaerobic conditions with half-lives reported up to 450 days [31]. Trifluralin is a widely-used pre-emergent soil-applied herbicide belonging to the Group 3 (dinitroanilines (DNAs)) chemical group [32], that effectively manages annual grass and broadleaf weeds in agricultural fields [33]. Trifluralin disrupts mitosis and microtubule assembly in plant cells by preventing tubulin polymerization, leading to growth inhibition and eventual plant death [34–37]. It has a high binding potential to soil [38], and a half-life reported in the range from 21 to 126 days [39]. Its low mobility, and low degradation rate enhance risk of carryover to susceptible rotational crops [40]. On the other hand, greater adsorption may reduce its bioavailability, reducing phytotoxicity. Predicting phytotoxicity *a priori* from chemical properties alone is fraught with uncertainty.

Despite occasional reports of herbicide-induced phytotoxicity under field conditions, little information is available about the toxicity thresholds level of residual herbicides for winter grain crops such as wheat, canola, chickpeas, lentil, lupin, and field peas [22]. Because phytotoxicity thresholds of specific herbicides for assessing plant-back risk are not readily available, reports of extractable herbicide residue levels in field soils are difficult to interpret, making plant back injury diagnosis for susceptible crops challenging. This limited knowledge is a major obstacle to developing management guidelines for preventing or avoiding crop phytotoxicity. This study evaluated phytotoxicity thresholds for major grain crops grown in southern Australia by exposing them to a range of priority residual herbicides, based on short-term dose response experiments. The aim was to identify phytotoxic concentrations of herbicides in the soil, which are directly relevant to crop growth inhibition. Outcomes from this research will also support more informed decisions for managing herbicide persistence and phytotoxicity problems, and planning of better cropping systems to minimise crop damage.

## Materials and methods

### Herbicide treatments, soil and plant species

Dose−response experiments were conducted to evaluate phytotoxicity of four herbicides that have different modes of action, namely clopyralid, pyroxasulfone, propyzamide and trifluralin in a washed sand soil for six common winter grain crops. The four priority herbicides were applied as their respective commercial products and recommended application doses (Table 1). Washed coarse sand was purchased (Perth Sand Supplies) and air dried before setting up the glasshouse experiment. The soil properties were measured by an external laboratory by standard methods [41] and the complete soil analysis results are presented in the Table 2. The washed sand was packed to a bulk density of 1.6 g cm^-3^ in the plastic pots.

**Table 1:** Label rates (g or ml ha^-1^) and commercial product names of herbicides applied to washed sand (bulk density- 1.6 g cm^-3^).

Commercial products	Product Label rates (g or ml ha^-1^)	Equivalent rate of active ingredient (g ha^-1^)
Clopyralid (Imtrade- Rally 300 g L^-1^)	250 ml ha^-1^	75
Pyroxasulfone (Bayer- Sakura Flow- 480 g L^-1^)	210 ml ha^-1^	100.8
Propyzamide (Imtrade EDGE WG- 900 g kg^-1^)	550 g ha^-1^	495
Trifluralin (Imtrade EC– 480 g L^-1^)	1250 ml ha^-1^	600

**Table 2:** Chemical and physical properties of the experimental soils.

Property	Washed sand
Organic carbon (%)	0.13
pH (CaCl_2_)	8.9
EC (dS m^-1^)	0.031
PBI	1.6
Effective CEC (cmol kg^-1^)	0.2
Clay (%)	0.6
Coarse sand (%)	51.8
Fine sand (%)	46.2
Silt (%)	1.4

*Note-* pH, -log of hydrogen ion activity; EC, Electrical Conductivity; PBI, Phosphorus Buffering Index; CEC, Cation Exchange Capacity

Canola (*Brassica napus* L. cv. ATR Bonito TT), chickpea (*Cicer arietinum* L. cv. PBA Striker), fieldpea (*Pisum sativum* L. cv. PBA Gunyag), lentil (*Lens culinaris* L. cv. PBA Hurricane XT), lupin (*Lupinus albus* L. cv. PBA Jurien) and wheat (*Triticum aestivum* L. cv. Scepter) were selected for testing as they are common varieties of these crops in Australia. High germination percentages of canola (99%), chickpea (100%), field pea (97%), lentil (96%), lupin (99%), and wheat (100%) seed were confirmed before the experiment.

### Experimental design and management

The dose−response experiment was conducted in a glasshouse at Murdoch University, Perth, Australia, that was maintained an average air temperature of 19°C and 36% relative humidity throughout the 28-day experiment. Eight concentrations of each herbicide were applied at rates equivalent to 0, 1/9, 1/6, 1/3, 1, 3, 6 and 9 times the rate specified on product labels for tolerant crops (Table 3). Note that we use this definition of ‘label rates’ when testing crops covered by the product label, and also non-tolerant crops that are not covered by the label. The soil concentrations of tested herbicides (μg kg^-1^ soil) were derived from their field application rates (g ha^-1^). To determine the amount of active ingredient needed in g ha^-1^, we estimated the herbicide required (a.i., μg kg^-1^) for inert sand based on its bulk density. The applied rate in units of mass per hectare was converted to concentration, assuming it was evenly distributed throughout the soil depth in the pots.

**Table 3:** Herbicide doses (target rate, g ha^−1^; active ingredient, a.i. μg kg^−1^ soil) applied for dose−response phytotoxicity assays.

Relative label rates	Clopyralid		Pyroxasulfone		Propyzamide		Trifluralin	
	g ha^ − 1^	μg kg^ − 1^	g ha^ − 1^	μg kg^ − 1^	g ha^ − 1^	μg kg^ − 1^	g ha^ − 1^	μg kg^ − 1^
0	0	0	0	0	0	0	0	0
1/9	8.3	6	11.2	7	55	30	66.7	42
1/6	12.5	8	16.8	10.5	82.5	50	100	63
1/3	25	17	33.6	21	165	100	200	125
1	75	50	100.8	63	495	310	600	375
3	225	150	302.4	189	1485	930	1800	1125
6	450	300	604.8	378	2970	1860	3600	2250
9	675	450	907.2	567	4455	2790	5400	3375

The selected herbicide rates for the dose-response study cover a broad spectrum, from sub-lethal to potentially toxic levels. This range enables a comprehensive assessment of the herbicide’s effects on crops, identifying both the minimum effective concentration and toxicity threshold. Lower rates capture subtle growth changes, while higher rates assess phytotoxicity and potential residual impacts on subsequent crops. This approach supports accurate modelling of dose-dependent responses and helps determine optimal rates for effective weed control with minimal carryover impact on non-target plants.

The experimental materials, including herbicide stock solutions and spiked soils with varying herbicide doses, were prepared according to the methods outlined in the previous study [42]. The soils were sealed in the bags and incubated in the glasshouse for 24 hours before planting of the tested crops.

A randomized complete block design was applied with 3 replicates of each treatment combination (i.e., 6 crops x 1 soil x 8 doses x 3 replications). Each treatment included three biological replicates, with two plants per pot, across eight herbicide concentrations, six crop species, and four herbicides. Eight doses were applied to determine the accurate toxicity thresholds values [43]. Following Burgos et al. [43], a broad dose range was prioritized over additional replication to ensure informative model fits. While the experiment was conducted once, the inclusion of three biological replicates ensures robustness and reproducibility of the results. Similar experimental designs have been used in previous herbicide bioassays [22,42,44,45].

Plastic pots (0.676 L); dimensions of 16 cm (H) x 6.5 cm x 6.5 cm (W) were each filled with 0.85 kg of soil. The experimental management, including plant watering, was conducted following the method described previously [42]. Four seeds were directly sown in each pot and covered with a plastic sheet to minimise moisture loss during germination. Seedling emergence was monitored daily for 7 days and recorded prior to thinning. The final emergence percentages were calculated relative to the number of seeds sown per pot. At 7 days after emergence, the seedlings were thinned to retain two uniform plants per pot. Throughout the experiment, pots were weighed daily to ensure soil moisture was consistently maintained at 80% of field capacity by applying deionized water to the sandy surface. To avoid nutrient deficiencies and promote healthy plant growth, a complete nutrient solution was administered on a weekly basis. Detailed information on the fertilizer application rates can be found in the S1 Table.

At 28 days after sowing, the plants were harvested and gently washed to remove soil from the roots. All intact plants were patted dry on paper towels after washing. The separated fresh roots and shoots were weighed, and maximum shoot lengths were manually measured with a ruler. Root length was measured with a digital image analysing system (WinRHIZO 2007d, Regent Instrument, Quebec, Canada). Root and shoot dry weight data were collected after materials were dried in an oven at 65°C for 48 hours to a constant weight.

### Data analysis

Plant data were converted to percentages relative to the means of untreated controls for shoot and root biomass and length to compare responses among species to each herbicide and concentrations by using the following equation [46],


$$\text{Inhibition}(\%)=\left(1-\frac{{\text{L}}_{\text{t}}}{\text{L}0}\right)\times \text{1}0\text{0}\%$$ (1)

where L_t_ represents the dry biomass and length of shoots and roots measured in the herbicide-treated soil and L_0_ represents those parameters in the nontreated soil conditions.

Seedling emergence data were analysed using a three-factor analysis of variance (ANOVA) to assess the effects of crop species, herbicide treatment, and their interaction. Tukey’s Honest Significant Difference (HSD) post hoc test was subsequently applied to identify pairwise differences among treatment combinations. To further quantify herbicide effects, dose−response curves were fitted for each crop-herbicide combination using the ‘drc’ package in R environment [47]. From these curves, the effective dose required to reduce crop emergence by 50% (ED_50_) was calculated.

To examine variation of growth inhibition, statistical analysis was performed by a three-way factorial analysis of variance (ANOVA) for 6 tested species under the 8 herbicide application doses with 4 herbicides using open-source statistical software R [48]. Two-way ANOVA was applied to explore the interaction effects of different herbicides and crops on the growth inhibition at the recommended application rate. The averages of growth reduction were compared by using Tukey’s HSD test and P-values were determined to evaluate the differences between combination of crops with dicot and monocots and herbicides which have a different mode of actions. QQ plots and fitted vs. residual plots were used post-hoc to assess assumptions regarding normality and homogeneity of variance.

Dose−response curves were fitted individually to each herbicide by crop combination using the ‘drc’ package [47] in R. From these the effective doses of herbicides (ED) required to reduce plant growth by 20% (ED_20_) and 50% (ED_50_) were calculated. Based on previous work [42], two dose response models were assessed for this study: the log-logistic equation:


$$Y=C+\frac{D-C}{\text{1}+\text{exp}\left(B\text{\textbackslash{} }\left(\text{log}\text{X}-\text{log}E\right)\right)}$$ (2)

and the Weibull type 2 equation:


Y=C+(D−C)exp(−exp(B(logX−logE))) (3)

where Y is the response of plant growth variable, C denotes the lower limit of the response when the dose X is very large; D is the upper limit when the dose X approaches 0. B is the slope around the point of inflection (ED_50_). The non-linear four parameter log-logistic model (Equation 2) was fitted to the shoot and root dry biomass and shoot length responses of all tested species. In some cases, the biomass and length of evaluated species are set to zero at maximum toxicity, which implies no shoot or root growth. Root lengths of all evaluated species were fitted by the three parameter Weibull type 2 model (Equation 3).

The best fitting dose response model was determined using the Akaike’s information criterion (AIC) [49,50]. The lack-of-fit test was also applied to evaluate p-values with the function of modelFit() (p value >0.05 means good fit of the model) [51]. Following the recommendation of [52], the actual biomass and length data were used to estimate ED values against the herbicide concentrations for each species and herbicide from dose−response curves.

## Results

### Emergence of crops affected by herbicides

**Fig 1:**
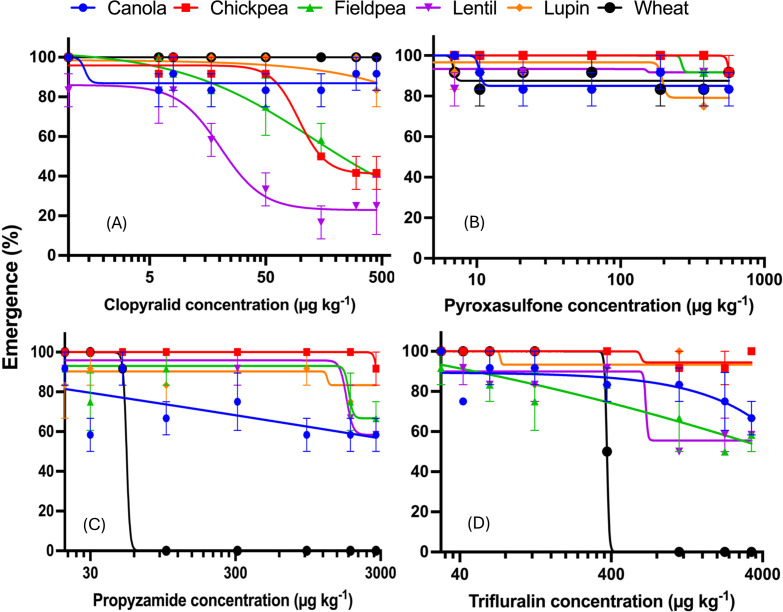
Effect of increasing soilborne concentrations of herbicides (A) clopyralid, (B) pyroxasulfone, (C) propyzamide, and (D) trifluralin on emergence of crops (compared to untreated control). Bars are standard error means of three replicates ± SE (n = 3).

A three-way ANOVA revealed significant interactions between herbicide treatments and crops species, indicating that herbicide effects on seedling emergence vary by plant species (p < 0.001) (S2 Table). Clopyralid notably reduced emergence in lentil (50% reduction at 29 μg kg^-1^) and also affected chickpea and fieldpea at higher concentrations (Fig 1A, Supplementary S3 Table). Emergence of all species was not significantly affected by pyroxasulfone (Fig 1B). Wheat was particularly sensitive to propyzamide and trifluralin, with complete emergence inhibition at 100 μg kg^-1^ and over 375 μg kg^-1^, respectively (Figs 1C and 1D, S3 Table).

### Herbicide by crop interactions at label rate

A three-way ANOVA revealed significant interactions between crop species, herbicide types, and application doses for all tested plant growth responses (S4 Table).

**Fig 2:**
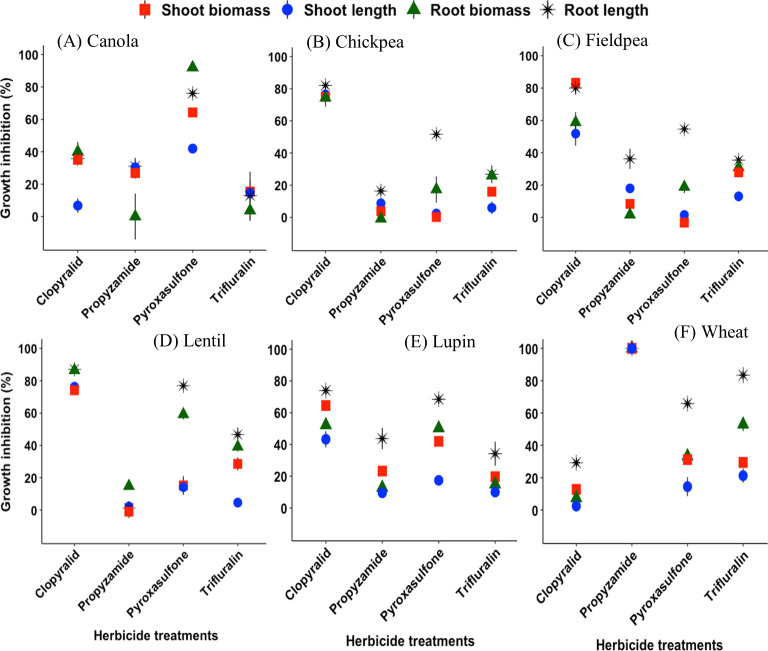
Shoot and root responses of crops to herbicides at the recommended application rates. Bars are standard error means of three replicates ± SE (n = 3).

Legumes (chickpea, fieldpea, lentil) were highly sensitive to clopyralid, showing severe shoot length and biomass inhibition, and even plant death at doses above the label rate (Fig 2). At the recommended rate of clopyralid, shoot length and biomass of legumes were more severely inhibited by 40−75 and 65−83%, respectively (p < 0.05) compared to other tested crops (Fig 2). In contrast, canola was less affected, with shoot inhibition at the label rate (50 μg kg^-1^) remaining below 40%, significantly lower than the inhibition observed in legumes (Fig 2A).

At the label rate of pyroxasulfone (63 μg kg^-1^), canola showed the highest shoot growth inhibition (42–64%), followed by lupin (42%), and wheat (30%) (Figs 2A, 2E, and 2F). However, root biomass was most reduced in canola (92%), lentil (59%) and lupin (50%), statistically more than in wheat, chickpea and fieldpea (~17–30%) (p < 0.05) (Fig 2). Root length was more affected than biomass at this dose.

At the propyzamide recommended rate (310 μg kg^-1^), wheat showed 100% shoot and root growth inhibition due to lack of emergence (Fig 2F).

Shoot growth was decreased by 2–30% at the recommended trifluralin dose (375 μg kg^-1^), but no significant response was observed across all species (Figs 2A–2F). However, wheat root length was severely inhibited (83.4%), much more than its biomass inhibition (51.6%) (Fig 2F) and significantly higher inhibition than other tested species (12.9–46.7%) (p < 0.05) (Fig 2F).

### Clopyralid toxicity thresholds

As expected, clopyralid significantly disrupted legumes growth, with shoot and root development being more severely inhibited than in wheat and canola (p < 0.05) (S1 Fig). Wheat exhibited high tolerance, showing less than 20% shoot reduction even at the highest clopyralid concentration (450 μg kg^-1^), while canola showed mild visual toxicity symptoms (slight twisting of the new leaves and pale-yellow leaves) but had uncertain ED values due to its relative resilience (Table 4). In contrast, legumes (chickpea, fieldpea, lentil, and lupin) were highly sensitive. The ED_50_ values for shoot growth in all legumes were below the recommended application dose (Table 4), except for lupin, which showed 50% inhibition only at doses above the label rate (S1c Fig).

**Table 4:** Estimated dose−response thresholds to clopyralid herbicide (μg kg^-1^soil) causing 50% (ED_50_) inhibition to shoot and root parameters of tested species.

Crops	Shoot biomass	Root biomass	Shoot length	Root length
	ED_50_ and 95% CI	ED_50_ and 95% CI	ED_50_ and 95% CI	ED_50_ and 95% CI
Canola	1240 (276-5570)	513 (17-15870)	134 (2e-18-1e + 22)	1738 (20-147430)
Chickpea	8 (5-13)	4 (2-8)	11 (7-15)	4 (2-5)
Fieldpea	3 (2-5)	27 (18-40)	16 (9-30)	7 (5-9)
Lentil	7 (4-12)	5 (2-14)	19 (16-24)	6 (3-12)
Lupin	18 (11-31)	67 (11-418)	179 (114-280)	9 (6-15)
Wheat	2994 (312-28738)	821 (342-1969)	690 (NaN)	274 (152-494)

*Note-* NaN means “Not a Number” as the value cannot be identified. NaN values in the confidence intervals (CI) indicate that the model was unable to estimate variability due to limited or highly variable data. Wide confidence intervals reflect high uncertainty in the ED estimates, likely caused by small sample sizes or poor model fit. Future studies should consider increasing sample size and improving dose-response design to reduce variability and improve confidence in ED estimates.

**Fig 3:**
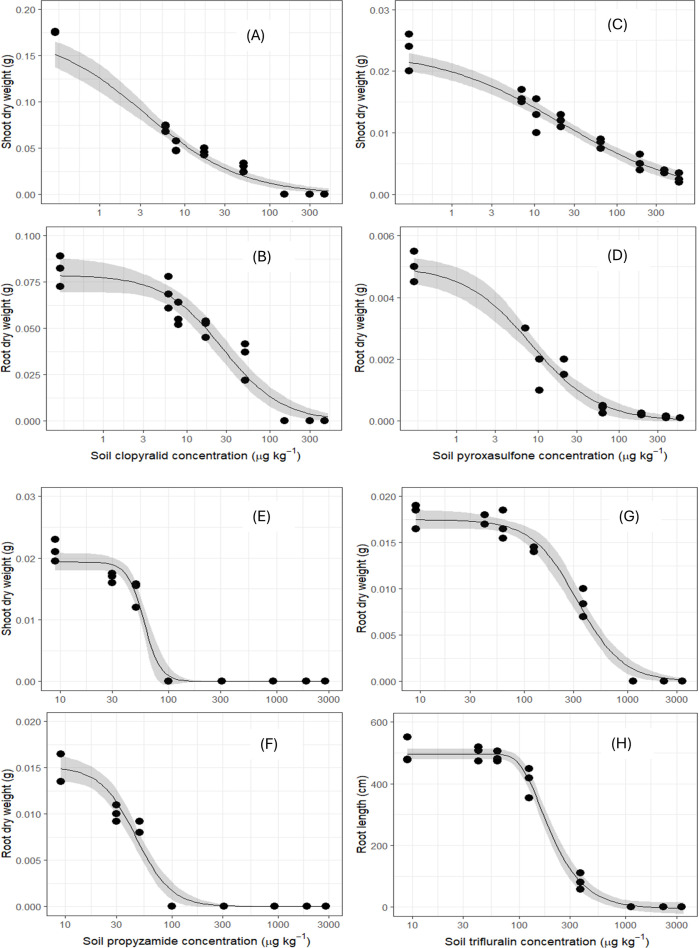
Log-logistic dose−response assays for shoot and root biomass of fieldpea (A and B) against clopyralid; canola (C and D) versus pyroxasulfone; wheat (E and F) versus propyzamide and wheat (G for root dry biomass and H for root length) against trifluralin. The shaded area represents the model fit at 95% confidence level. The most sensitive species to different herbicides are presented in this figure.

The required amount of clopyralid causing 20% inhibition in legume crops occurred at the second lowest concentration applied (8 μg kg^-1^) (S6 Table). Fieldpea was particularly vulnerable, with an ED_50_ for shoot biomass estimated at just 3 μg kg^-1^ soil, lower than the minimum clopyralid concentration tested (6 μg kg^-1^ soil) (Table 4; Fig 3A).

Root growth inhibition in legumes ranged from 40−85% at the label rate (Figs 1B–1E), with ED_50_ values for root length below 10 μg kg^-1^ soil (Table 4). Chickpea and lentil showed 50% root biomass inhibition at only 4−5 μg kg^-1^, while fieldpea and lupin had higher ED_50_ values for root biomass (27 and 67 μg kg^-1^), indicating slightly greater root tolerance compared to shoot responses (Table 4, Figs 3A and 3B).

### Pyroxasulfone toxicity thresholds

Canola was the most sensitive crop to pyroxasulfone, showing significant shoot biomass (30%) and shoot length (20%) reductions even at the lowest concentration (7 μg kg^-1^ dry soil) (S1a and S1c Figs). Its ED_50_ values for shoot biomass and length were 21 and 93 μg kg^-1^, respectively, which was significantly lower than other crops (p < 0.05) (Table 5, Fig 3C).

**Table 5:** Estimated dose−response thresholds to pyroxasulfone herbicide (μg kg^-1^soil) causing 50% (ED_50_) inhibition to shoot and root parameters of tested species.

Crops	Shoot biomass	Root biomass	Shoot length	Root length
	ED_50_ and 95% CI	ED_50_ and 95% CI	ED_50_ and 95% CI	ED_50_ and 95% CI
Canola	21(14-31)	8 (6-11)	93 (73-117)	6 (4-9)
Chickpea	1497 (0.9-2493400)	808 (319-2046)	2125 (66-67996)	53 (37-75)
Fieldpea	4147 (247-61667)	525 (278-989)	2474 (502-12190)	34 (22-52)
Lentil	3471 (480-25099)	27 (17-44)	671 (531-847)	12 (8-18)
Lupin	159 (77-330)	66 (27-164)	297 (225-392)	17 (10-28)
Wheat	330 (135-807)	295 (140-621)	347 (291-414)	22 (16-30)

Root growth inhibition (25−40%) at the lowest dose was similar across canola, lentil, and wheat (S1b Fig), but canola root biomass and length were highly sensitive with ED_50_ values of just 8 and 6 μg kg^-1^, respectively. These values were markedly lower than those of other species, which ranged from 27–808 μg kg^-1^ for root and for shoots inhibition between 12–53 μg kg^-1^ (Table 5, Fig 3D). All evaluated crops experienced over 50% root elongation reduction, with no significant differences among species (p < 0.05) (Figs 1A–1F).

Notably, the estimated ED_20_ of canola for both shoot and root growth were below the minimum application rate, while chickpea and fieldpea showed relative tolerance, making their ED_20_ estimates less reliable (S7 Table).

### Propyzamide and trifluralin toxicity thresholds

Wheat was the most susceptible crop to both propyzamide and trifluralin, which are primarily used for grass weed control. Even at low concentrations (30 μg kg^-1^ for propyzamide and 42 μg kg^-1^ for trifluralin), wheat shoot biomass was reduced by 20 and 11.5%, respectively, compared with untreated plants (S1a Fig). At higher trifluralin concentrations over the label rate, all tested species showed significant shoot growth inhibition (S1a and S1c Figs). Wheat had the lowest ED_50_ values for shoot growth (Fig 3E), which were 10–52 times and 1.3–7 times lower than those of other tested crops (Tables 6 and 7).

Root length of wheat was particularly sensitive to trifluralin, with a 67% reduction at the lowest dose, significantly more than other evaluated crops (−1 to 0.4%) (p < 0.05) (S1d Fig). The ED_50_ values for wheat root length and biomass under propyzamide exposure were 22 and 46 μg kg^-1^ soil, respectively (Table 6 and Fig 3F), up to 67 times lower than for other tested species. For trifluralin, ED_50_ values for wheat root biomass and length were 347 and 194 μg kg^-1^ soil, respectively (Table 7, Figs 3G and 3H), still lower than those of other tested crops (Table 7). Overall, the ED_20_ values for wheat root and shoot growth were below these herbicides label rates, highlighting its high sensitivity (S8 and S9 Tables).

**Table 6:** Estimated dose-response thresholds to propyzamide herbicide (μg kg^-1^ soil) causing 50% (ED_50_) inhibition to shoot and root parameters of tested species.

Crops	Shoot biomass	Root biomass	Shoot length	Root length
	ED_50_ and 95% CI	ED_50_ and 95% CI	ED_50_ and 95% CI	ED_50_ and 95% CI
Canola	592 (485-722)	748 (534-1047)	955 (434-2099)	525 (424-649)
Chickpea	3062 (2551-3674)	2155 (1788-2597)	4528 (1871-10959)	714 (598-852)
Fieldpea	2703 (2307-3167)	2443 (1812-3294)	3291 (2473-4381)	638 (432-943)
Lentil	2431 (2073-2851)	1666 (1483-1871)	3024 (2710-3374)	1469 (1305-1654)
Lupin	1199 (828-1736)	605 (458-801)	1025 (938-1122)	370 (292-470)
Wheat	59 (54-65)	46 (39-54)	61 (50-75)	22 (16-30)

**Table 7:** Estimated dose−response thresholds to trifluralin herbicide (μg kg^-1^ soil) causing 50% (ED_50_) inhibition to shoot and root parameters of tested species.

Crops	Shoot biomass	Root biomass	Shoot length	Root length
	ED_50_ and 95% CI	ED_50_ and 95% CI	ED_50_ and 95% CI	ED_50_ and 95% CI
Canola	1032 (803-1262)	1014 (810-1270)	1391 (1070-1807)	828 (664-1033)
Chickpea	1154 (837-1471)	824 (607-1119)	1734 (1426-2108)	601 (515-702)
Fieldpea	681 (484-879)	731 (563-950)	938 (786-1119)	492 (432-561)
Lentil	613 (397-829)	379 (179-801)	1104 (1034-1178)	295 (163-533)
Lupin	3222 (1843-4601)	6273 (1995-19723)	4604 (3151-6728)	625 (497-786)
Wheat	475 (278-811)	347 (264-456)	479 (275-833)	194 (161-235)

## Discussion

Residual herbicides, even in low concentrations, can significantly affect crop performance. This study found that one or more herbicides had the potential to cause significant phytotoxic effects in all crops at concentrations below recommended application rates and below extractable concentrations detected in a recent field survey of pre-sowing herbicide residues in field soils around Australia [22]. These results suggest the risk of early crop damage and yield loss due to residual herbicides in very light-textured soils. This expands on our previous research that demonstrated that soil residual diuron herbicide exhibit varying levels of phytotoxicity across different crops, such as canola, chickpea, and wheat [42]. The present findings provide a basis for assessing plant back risk of common winter crops in light-textured soils by identifying ED_50_ values for four commonly used herbicides with different modes of action.

The present ED50 values represent a worst-case scenario for herbicide mobility and bioavailability, since they were obtained using a single, highly permeable soil type, a sand with low organic matter. While this approach aligns with established risk assessment practices, soil properties such as clay content, organic matter, pH, microbial activity, and structure significantly influence herbicide sorption, persistence, and phytotoxicity [5,53]. The fate of herbicides in soil is primarily determined by sorption and degradation processes, which are influenced by soil characteristics such as texture, mineralogy, and organic matter contents [54–56].

In the present study, we observed that each of the four herbicides tested has potential carryover toxicity on susceptible rotational crops under worse-case scenarios where bioavailability is at a maximum (i.e., in sand where organic matter and clay content are negligible). While this leads to low ED values, it could prove useful for extrapolating results to other soils by providing a worst-case baseline. The low ED values indicate that the plant is highly sensitive to herbicides, which is particularly relevant for sandy soils due to their low adsorption ability. This baseline can serve as a reference point for understanding how herbicides might behave in more retentive soils. Adjusting the ED values according to expected partitioning onto soils (for example, by considering the soil adsorption coefficient, Kd), can help account for differences in soil properties. While effective dose (ED) values can be extrapolated across soil types using partition coefficients (Kd) [57] there is currently insufficient evidence to assume linear and predictable relationship to soil properties [58]. Future research is needed on a broader range of soil types to derive such relationships. By incorporating these adjustments, we can more accurately predict herbicide behaviour in different soils, thus improving risk assessment and guiding safer agricultural practices.

### The plant back risk of clopyralid on the tested crops

Clopyralid targets broadleaf weeds, therefore plant back risks for legume crops are expected. Similar to previous studies, our research showed a severe impact of clopyralid on grain legumes compared to wheat and canola. Below the label application rate, clopyralid suppressed lentil seed emergence by 50% (Fig 2A). Higher soil concentrations of clopyralid further delayed grain legumes emergence, possibly due to auxin-like effects, which may disturb normal seedling growth and lead to improper establishment [12,24,59,60].

The present findings, consistent with previous research, indicate that clopyralid severely impacts the growth of legume cultivars like chickpea, fieldpea, and lentil, highlighting the risk of plant back injuries in susceptible grain legumes even at low concentrations [12]. This is reflected in the recommended plant back interval for legumes of 9 months to 2 years [61]. Despite aligning with previous findings that lupin is less affected by clopyralid (Congreve and Cameron [62], Peirce et al. [63]), the specific reasons behind lupin’s tolerance to this herbicide remain unclear in the literature.

Of greater concern were the ED_20_ values we found for grain legumes that were below the maximum residue load of 6 μg kg^-1^ that was detected in Australian field soils in the upper 0–10 cm depth [22]. Twisting of new shoots and stems of grain legumes (except for lupin) were observed at a clopyralid dose of 6 μg kg^-1^. This is likely due to herbicide’s mode of action including auxin imbalanced and increases abscisic acid (ABA) production, leading to plant senescence and increased ethylene levels, ultimately uncontrolled and inhibited growth of the plants [24,64]. Juras and Irvine [65] reported no adverse impacts to chickpea, lentil and field beans 11 months after clopyralid application at 100 g a.e ha^-1^ which they attributed to high soil moisture and temperatures promoting soil microbial degradation [66]. In contrast, the dry summer fallow, a trend to earlier sowing and declining growing season rainfall in the Western Australia wheatbelt may increase risks in a rotational legume crops. Moreover, clopyralid’s high solubility and low adsorption capacity allows it to penetrate more deeply into the sub-surface soil with low microbial activity, enhancing persistence [62,67].

Rose et al. [22] found higher ED_50_ values for lupin than in this study although suppression of lupin growth was observed in both studies. Sandy soil type likely explains the lower values in this study. The present findings are consistent with previous studies by Lindenmayer [68] and Bukun et al. [69], which suggest that the adsorption behaviour of clopyralid is significantly and positively correlated with the clay, organic matter and silt contents of soils. Consequently, the low levels of these properties in this washed sand soil may lead to reduce herbicide adsorption and lower biodegradation, resulting in increased plant phytotoxicity.

Therefore, this study supports label recommendations that restrict crop rotation for grain legumes after higher rates of clopyralid application, particularly in sandy soils, while wheat and canola are relatively tolerant to clopyralid and can be planted as rotational crops.

### The plant back risk of pyroxasulfone on the tested crops

This study shows that canola is the most susceptible crop to pyroxasulfone, which is mainly used to control grass weeds and some young dicots. The estimated ED_50_ of canola shoot biomass response (21 μg kg^-1^) was below the label rate which is consistent with the result of Walsh et al. [70] (ED_50_ = 40 g ha^-1^ or ~25 μg kg^-1^), and reflects translocation of chemical from the roots to the shoots of the plants [64]. The ED_50_ for canola from this study was lower than pyroxasulfone residues (27 μg kg^-1^) detected in the Australian field soils, according to soil-survey results in 2016 [22].

As reported by Congreve and Cameron [61], plant back injuries may occur in canola, wheat, chickpea, fieldpea, and lupin within 9 months after applying pyroxasulfone (70 g ha^-1^). In canola plants treated with higher concentrations of pyroxasulfone, only cotyledons emerged, and no additional leaves appeared due to a decrease in fatty acid synthesis inhibiting cell division and expansion at the new shoot and root growth point [25,71,72]. The roots of all tested species are more sensitive to pyroxasulfone than shoots, not only in terms of lower ED_20_ (S7 Table) and ED_50_ values, but also their root length inhibition, which varied from 20 to 40% at the lowest concentration (Table 7 and S1d Fig).

Based on research by Yamaji et al. [73], plumules or mesocotyls in direct contact with soil containing residual pyroxasulfone herbicide absorb the substance primarily through their roots, leading to a more pronounced effect on the roots compared to the shoots. Wheat and fieldpea were the most tolerant to pyroxasulfone among the evaluated species. Similar to this study, previous researchers have reported the tolerance of legumes and wheat to pyroxasulfone in terms of their overall survival rates (>95%) and shoot biomass inhibition under 10% at field application rate (100 g ha^-1^) [70].

Although 30% decrease in wheat shoot growth occurred at the label rate in this experiment, wheat yield may not be inhibited due to recovery in later growth stages. For example, Timothy and Larry [74] reported that there was no yield reduction in wheat plants despite temporary injury at 120 g pyroxasulfone ha^-1^. Thus, the results of this study indicate that pyroxasulfone can be used safely in wheat crops to control grass weeds, which is similar to recommendations in previous studies [75,76].

### The plant back risk of propyzamide and trifluralin on the tested crops

Wheat was the most sensitive to propyzamide and trifluralin herbicides as these herbicides are predominantly used to control grass weed. The emergence of wheat was extremely sensitive to propyzamide as 50% inhibition occurred below the label rate (Fig 1C). Seedling emergence of wheat was completely retarded by propyzamide and trifluralin concentrations at 100 μg kg^-1^ and over 375 μg kg^-1^, respectively (Figs 1C and 1D). Due to binding of propyzamide to the microtubule protein tubulin, susceptible wheat crops experience mitosis retardation and loss of microtubule structure, inhibiting the growth of shoots and roots [77]. Wheat and oat did not emerge at trifluralin doses above 300 μg kg^-1^ due to interrupting meristematic cell division through the absorption of the hypocotyl and radicles [78–80].

As no shoots emerged at the field application rate of propyzamide, it is possible that propyzamide inhibits cell division at the root tips, potentially halting wheat shoot development. This result agrees with earlier studies by Corre-Hellou and Crozat [81], Rouchaud et al. [82] and Vouzounis and Americanos [83], which found that propyzamide herbicide inhibited shoot growth of barley, winter wheat, and oats. Wheat exhibited the smallest ED_50_ (22–46 μg kg^-1^) for root length and biomass response to propyzamide among the tested species. ED_20_ for root inhibition of wheat (12.6–27 μg kg^-1^) was estimated at lower than minimum propyzamide dose (30 μg kg^-1^) (S8 Table). The lowest dose of propyzamide reduced wheat root length by 67%, leading to swelling and clubbing of root tips by disrupting microtubule function during mitotic cell division, potentially causing root tip cell death, with greater root damage than shoot damage in sensitive species likely due to root absorption and inhibited cell elongation [84,85].

Trifluralin also had less impact on the shoots of all tested species than roots, due to limited translocation and activity in leaves, fruit, or seeds [86] but continued disruption of mitosis in the roots [87]. In this study, ED_50_ values of trifluralin for shoot biomass in wheat and lupin were 475 μg kg^-1^ and 3222 μg kg^-1^, respectively. These values were 1.2 and 2.8 times lower than the ED_50_ values (559 and 9034 μg kg^-1^) estimated by Rose et al. [22], likely due to the lower organic carbon content (0.13%) in the current experimental soil, which increased trifluralin bioavailability and plant back constraints compared to soil with 1% organic carbon in Rose et al. [22] study.

Root length of wheat was extremely impacted by trifluralin since its ED_50_ values are relatively low (Table 7). Root elongation of wheat was inhibited by 83% at the trifluralin field application dose, while other species were decreased less than 50%. The ED_50_ of wheat root length was 2.5-fold lower than the dose of trifluralin required to reduce 50% shoot length. The impact of trifluralin on the roots over shoots were reported by earlier researchers, Almeida and Rodrigues [88], who assessed trifluralin residues in different plant parts and noted that residues were obvious in the roots rather than shoots.

A maximum concentration of trifluralin residues was observed in Australian field soils (590 and 5345 μg kg^-1^) in 2015 and 2016 [22], as well as in other soils around the world in the range of 200 to over 1200 μg kg^-1^ [89–93]. ED_50_ values for wheat growth response (194–479 μg kg^-1^) from this study were lower than these soil trifluralin residues. However, the potential plant back risk of wheat crops due to trifluralin carryover effects in field soils may vary significantly depending on soil type and the location of trifluralin within the soil. For example, Chauhan et al. [94] reported that the highest concentration of trifluralin residue is likely found in the inter-row areas and near the soil surface due to soil displacement during sowing and restricted movement through the soil profile. Deep sowing of the crop may mitigate exposure to higher concentrations of trifluralin in the top soil, with the bioavailability of its residues potentially decreasing over time and differing significantly across soil types [95]. Therefore, we recommend caution in situations where sandy soil types are present, especially when considering rotation restrictions for cereal crops following the application of propyzamide and trifluralin herbicides over short periods.

### Limitations and future directions

We recognize that the use of a single, highly permeable soil type, a sand with low organic matter, represents a worst-case scenario for herbicide mobility and bioavailability. While this approach is commonly employed in risk assessment to ensure conservative estimates, the variability of soil types and field conditions will produce different and mostly lower soil solution herbicide concentrations than the equivalent rate applied to sand. Therefore, site-specific soil characteristics such as soil texture, pH, organic matter content, and microbial activity should be considered when interpreting and applying these findings in practical settings. In addition, our results provide some indication of potential injury to seedlings, but the effect on final crop yield will likely be dependent on a range of seasonal and edaphic factors. When setting ED_50_ values for varied soil types and crop species, the reproducibility of values can be tested by repetition of trials. Alternatively, as in the present study the experimental design aligns with establishes herbicide bioassay practices [22,44,45]. Burgos et al. [43] suggest that a broader dose range can be more informative than additional replication when model fitting is adequate. Nevertheless, additional dose-response experiments would provide increasing confidence in the reliability of ED_x_ values for different environmental conditions.

For practical weed management on farms, many farmers rotate herbicides among different modes of action and use binary or tertiary herbicide combinations to control weeds effectively and prevent weed resistance [96,97]. Even though mixtures of herbicides are widely used, the consequences of combined carryover effects on plant back injury risk of rotational crops are not well understood. Therefore, this knowledge gap is another piece of information that is required for minimising and avoiding plant back injury to crops from herbicide residues.

## Conclusions

This study evaluated the responses of canola, chickpea, fieldpea, lentil, lupin and wheat to the herbicides clopyralid, pyroxasulfone, propyzamide and trifluralin. Grain legumes and canola were most sensitive to clopyralid and pyroxasulfone, respectively, while wheat was most affected by propyzamide and trifluralin. Trifluralin severely inhibited root length across all crops, with wheat emergence completely retarded below label rates of propyzamide and trifluralin, and lentil highly sensitive to clopyralid field application rate. The relative phytotoxicity of herbicides varied by crop: for wheat, propyzamide > trifluralin > pyroxasulfone > clopyralid; for canola, pyroxasulfone > trifluralin > propyzamide > clopyralid; and for grain legumes, clopyralid > trifluralin > propyzamide > pyroxasulfone. The approximate ED_20_ values of clopyralid for legumes (<6 μg kg^-1^) and ED_50_ values of pyroxasulfone except from shoot length for canola (6−21 μg kg^-1^) were lower than the estimated residues load (6 μg kg^-1^) and (27 μg kg^-1^), respectively, found in Australian field soils. Similarly, trifluralin ED_50_ values for wheat were below global soil residue levels (~200−1200 μg kg^-1^), indicating a potential risk of phytotoxicity to subsequent non-target crops, but several other factors that will influence whether toxicity is realised. Glasshouse bioassays proved useful for estimating plant back injury risk but field validation remains essential. These findings highlight the importance of herbicide selection and crop rotation planning. Farmers should avoid planting sensitive crops such as lentil or canola in fields recently treated with herbicides that have high persistence and phytotoxicity. Herbicide applications should always follow label guidelines to ensure safe use. While glasshouse trials offer insights into early crop responses, they don’t necessarily reflect final yield outcomes. Future research should focus on refining ED_10_ and ED_20_ values for crop emergence in susceptible species through targeted studies with selected herbicides. It should also investigate herbicide persistence across various soil types and environmental conditions, as well as the impact of herbicide mixtures on rotational crops. These efforts are essential to minimize plant loss and ensure successful crop establishment and productivity.

## Supporting information

S1 Fig Relative shoot and root inhibition (% compared to untreated control) versus various herbicide concentrations, d1 is the lowest tested rate and d7 is the maximum application rates for each herbicide (the application rates (μg kg^-1^ soil) are shown in the Table 4.3). (a) shoot dry weight inhibition, (b) root dry weight inhibition, (c) shoot length inhibition and (d) root length.
(DOCX)

S1 Table Mixture of nutrient solution. (DOCX)

S2 Table Three factor analysis of variance results (mean squares and significance) for the effect of crop species, herbicides, herbicide doses and their interactions on crop emergence. (DOCX)

S3 Table Estimated dose−response thresholds to clopyralid herbicide (μg kg^-1^soil) causing 50% (ED_50_) inhibition to emergence of crops. (DOCX)

S4 Table Three factor analysis of variance results (mean squares and significance) for the effect of crop species, herbicides, herbicide doses and their interactions on crop growth responses. (DOCX)

S5 Table Two factor analysis of variance results (mean squares and significance) for the effect of crop species, herbicides and their interactions on crop growth responses at label rate. (DOCX)

S6 Table Estimated dose-response thresholds to clopyralid herbicide (µg kg^-1^soil) causing 20% (ED_20_) inhibition to shoot and root parameters of tested species at 4 weeks after sowing. (DOCX)

S7 Table Estimated dose-response thresholds to pyroxasulfone herbicide (µg kg^-1^soil) causing 20% (ED_20_) inhibition to shoot and root parameters of tested species at 4 weeks after sowing. (DOCX)

S8 Table Estimated dose-response thresholds to propyzamide herbicide (µg kg^-1^soil) causing 20% (ED_20_) inhibition to shoot and root parameters of tested species at 4 weeks after sowing. (DOCX)

S9 Table Estimated dose-response thresholds to trifluralin herbicide (µg kg^-1^soil) causing 20% (ED_20_) inhibition to shoot and root parameters of tested species at 4 weeks after sowing. (DOCX)

S1 Data Supporting data_paper 2. (XLSX)
