# Supplementary material for: Herbicide dose-response thresholds in sands to assess the risk of non-target damage to winter grain crops
Source: PLoS One. 2025 Aug 21;20(8):e0330225. doi: 10.1371/journal.pone.0330225 (PMC12370053; doi:10.1371/journal.pone.0330225)
Supplement: S1 Fig — (a) shoot dry weight inhibition, (b) root dry weight inhibition, (c) shoot length inhibition and (d) root length. (DOCX) [file pone.0330225.s001.docx]

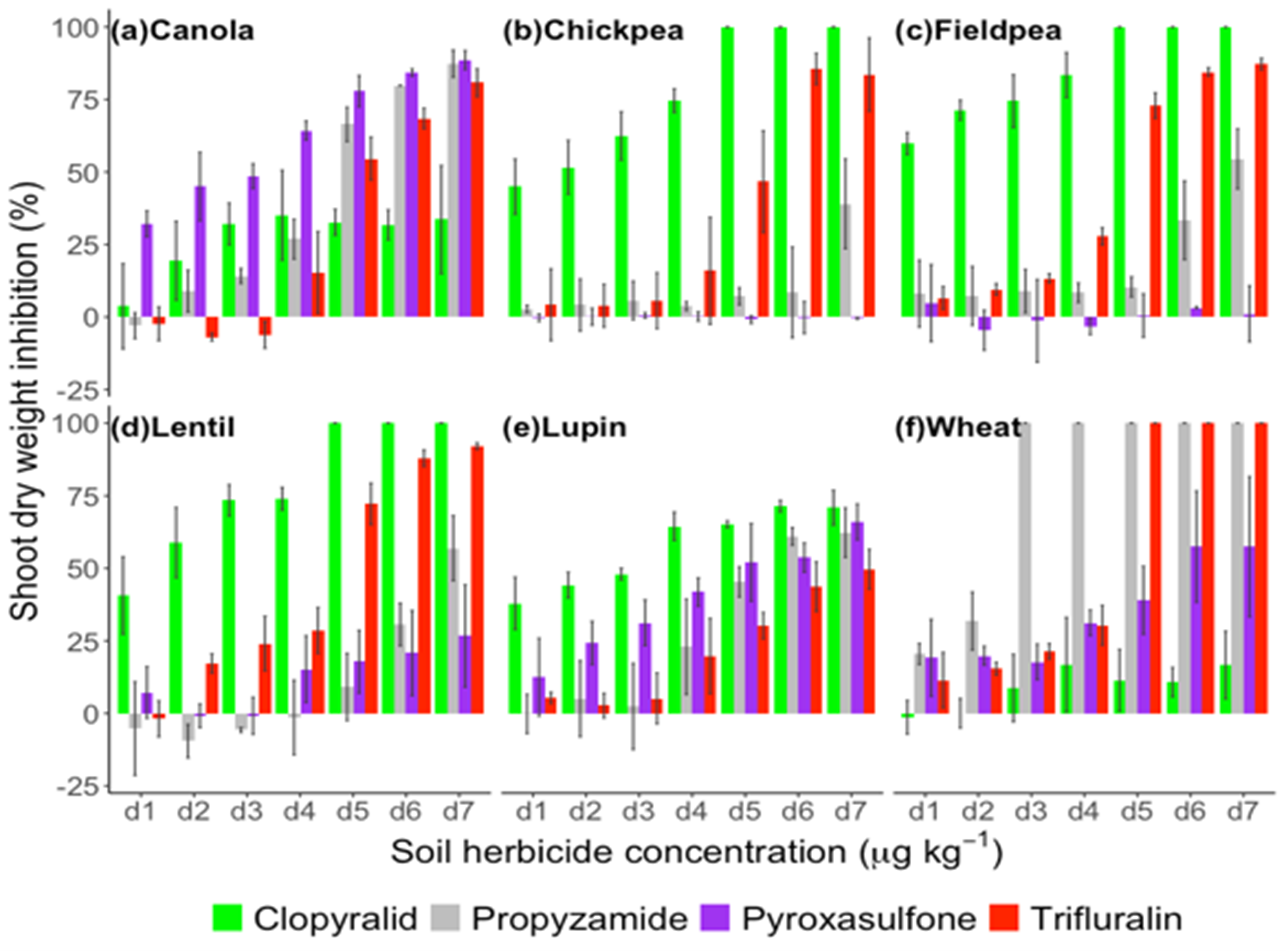


(a)


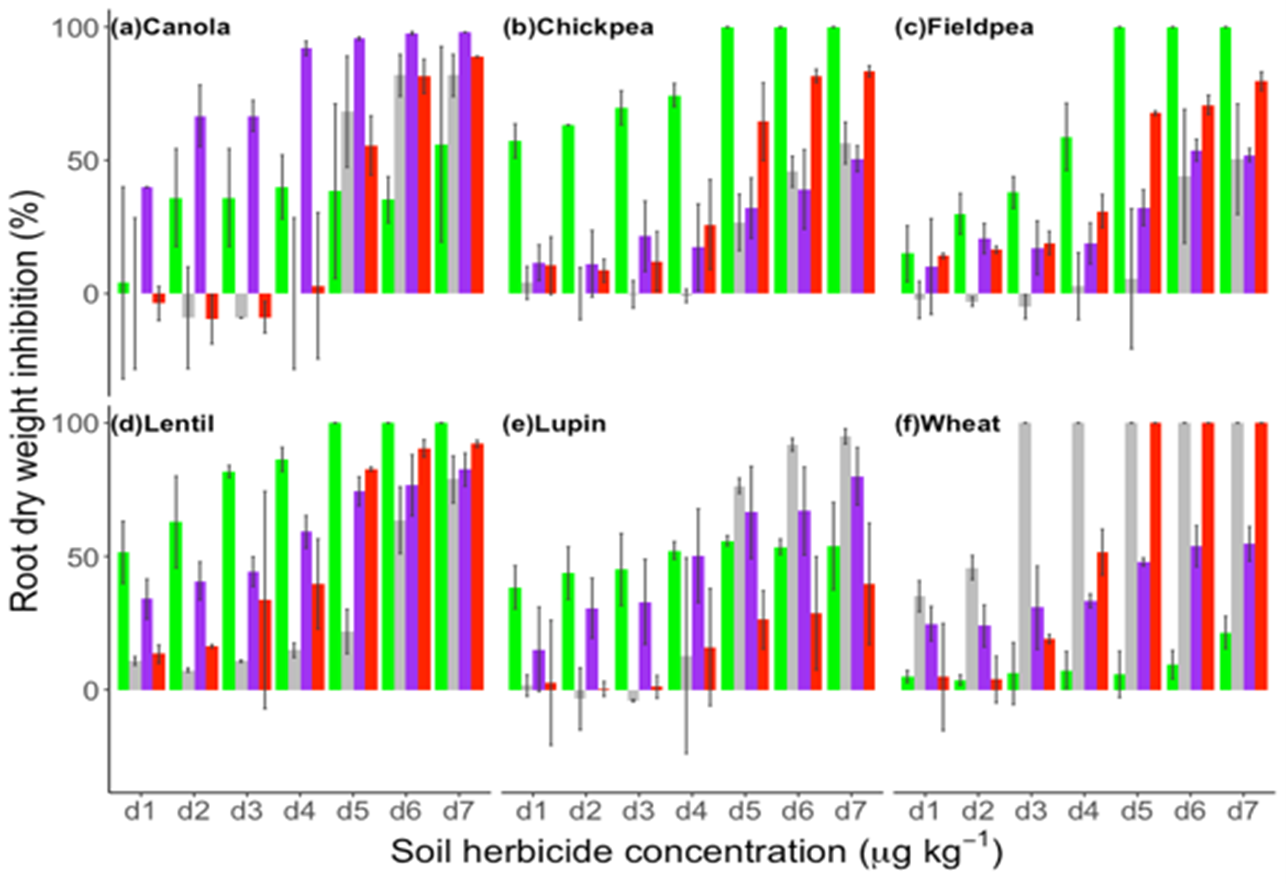


(b)


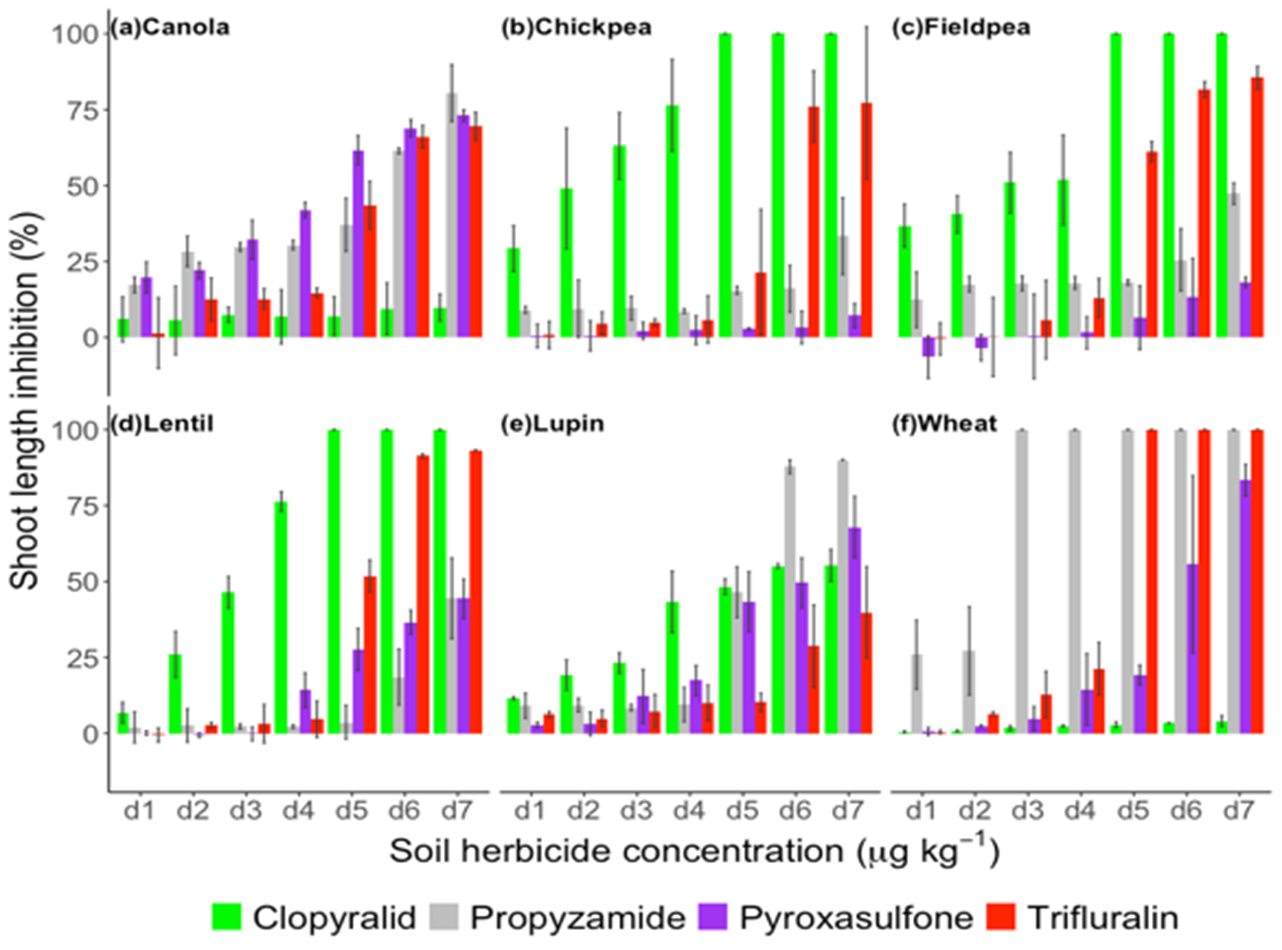


(c)

(d)


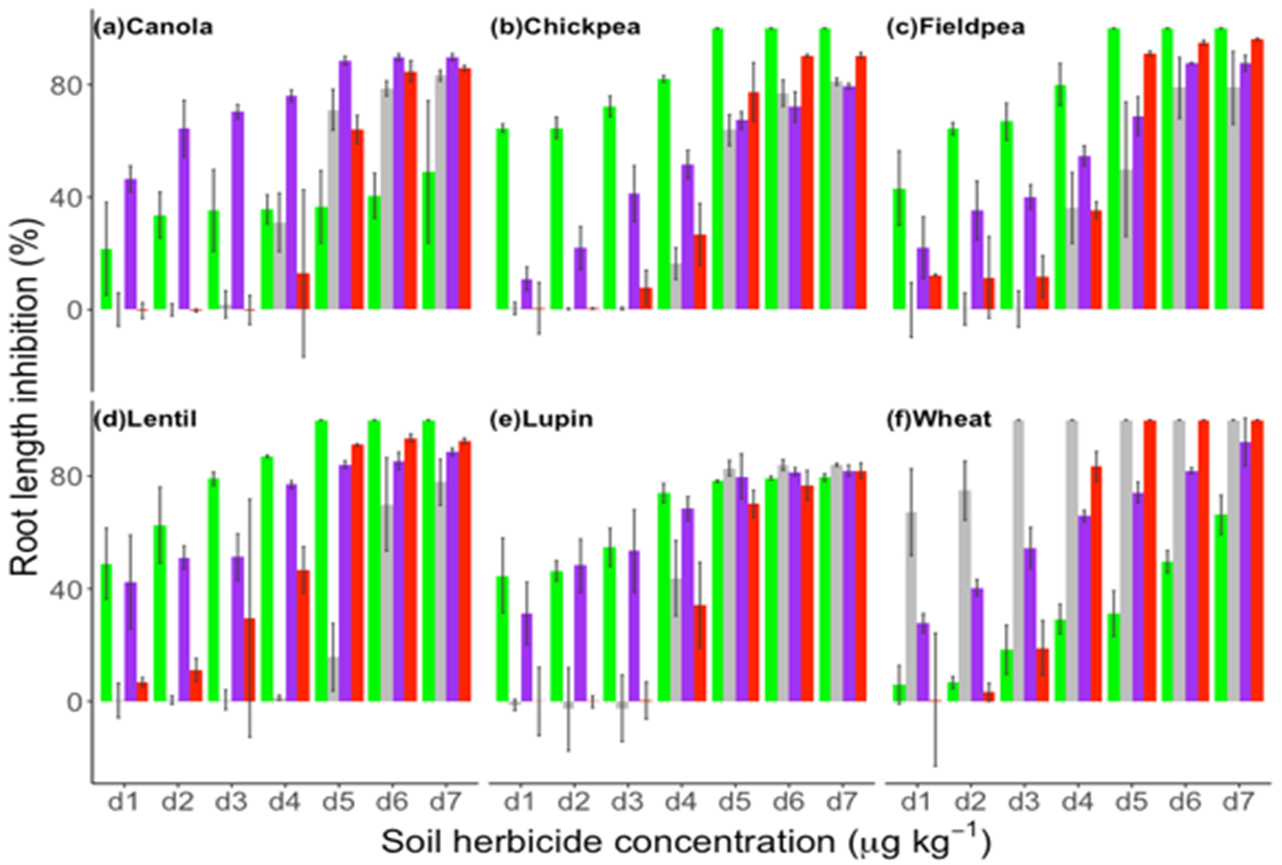


**S1 Fig.** Relative shoot and root inhibition (% compared to untreated control) versus various herbicide concentrations, d1 is the lowest tested rate and d7 is the maximum application rates for each herbicide (the application rates (μg kg^-1^ soil) are shown in the Table 4.3). (a) shoot dry weight inhibition, (b) root dry weight inhibition, (c) shoot length inhibition and (d) root length inhibition. Bars are standard error means of three replicates ± SE (n=3).
