# Supplementary material for: Herbicide dose-response thresholds in sands to assess the risk of non-target damage to winter grain crops
Source: PLoS One. 2025 Aug 21;20(8):e0330225. doi: 10.1371/journal.pone.0330225 (PMC12370053; doi:10.1371/journal.pone.0330225)
Supplement: S2 Table — (DOCX) [file pone.0330225.s003.docx]

| **S2 Table.** Three factor analysis of variance results (mean squares and significance) for the effect of crop species, herbicides, herbicide doses and their interactions on crop emergence.   \| **Source** \| **DF** \| **Emergence** \| **F value** \| **Pr (>F)** \| \| --- \| --- \| --- \| --- \| --- \| \| Block \| 2 \| 327 \| 2.35 \| 0.1 ^ns^ \| \| Crop (C) \| 5 \| 7223 \| 51.97 \| <2e-16 *** \| \| Herbicide (H) \| 3 \| 6084 \| 43.77 \| <2e-16 *** \| \| Dose (D) \| 7 \| 9612 \| 69.16 \| <2e-16 *** \| \| C x D \| 35 \| 778 \| 5.6 \| <2e-16 *** \| \| C x H \| 15 \| 5959 \| 42.87 \| <2e-16 *** \| \| H x D \| 21 \| 733 \| 5.27 \| 3.38e-12 *** \| \| C x H x D \| 105 \| 794 \| 5.71 \| <2e-16 *** \|   all effects were highly significant (P < 0.001 by Tukey’s HSD test).  *Note-* DF denotes degrees of freedom, *Note-* DF denotes degrees of freedom,  Emergence- Emergence of crops. |
| --- | --- | --- | --- | --- | --- | --- | --- | --- | --- | --- | --- | --- | --- | --- | --- | --- | --- | --- | --- | --- | --- | --- | --- | --- | --- | --- | --- | --- | --- | --- | --- | --- | --- | --- | --- | --- | --- | --- | --- | --- | --- | --- | --- | --- | --- |
