# Supplementary material for: Herbicide dose-response thresholds in sands to assess the risk of non-target damage to winter grain crops
Source: PLoS One. 2025 Aug 21;20(8):e0330225. doi: 10.1371/journal.pone.0330225 (PMC12370053; doi:10.1371/journal.pone.0330225)
Supplement: S3 Table — (DOCX) [file pone.0330225.s004.docx]

**S3 Table.** Estimated dose−response thresholds to clopyralid herbicide (μg kg^-1^soil) causing 50% (ED_50_) inhibition to emergence of crops

| **Herbicide** | **Crops** | **ED_50_**  **(μg kg^-1^soil)** | **Lower 95% CI** | **Upper 95% CI** |
| --- | --- | --- | --- | --- |
| Clopyralid | Canola | 5e+09 | 1e+07 | 2e+12 |
|  | Chickpea | 235 | 158 | 349 |
|  | Fieldpea | 219 | 103 | 466 |
|  | Lentil | 29 | 14 | 60 |
|  | Lupin | 585 | 97 | 3511 |
|  | Wheat | NaN | NaN | NaN |
| Pyroxasulfone | Canola | 1819 | 70 | 47228 |
|  | Chickpea | 1326 | 0.4 | 4351800 |
|  | Fieldpea | 7916 | 93 | 672157 |
|  | Lentil | 11455 | 2e-10 | 5e+17 |
|  | Lupin | 3664 | 343 | 39093 |
|  | Wheat | 3385 | 55 | 206572 |
| Propyzamide | Canola | 801 | 2e-04 | 3e+09 |
|  | Chickpea | 4913 | 648 | 37226 |
|  | Fieldpea | 4305 | 1936 | 9576 |
|  | Lentil | 3203 | 2170 | 4729 |
|  | Lupin | 3e+09 | 2e+08 | 5e+10 |
|  | Wheat | 68 | 16 | 293 |
| Trifluralin | Canola | 78908 | 280 | 22241000 |
|  | Chickpea | 6e+06 | 5e-04 | 7e+16 |
|  | Fieldpea | 5321 | 1265 | 22377 |
|  | Lentil | 4790 | 1665 | 13788 |
|  | Lupin | 3e+09 | 5e+08 | 2e+10 |
|  | Wheat | 375 | 375 | 375 |
