# Supplementary material for: Herbicide dose-response thresholds in sands to assess the risk of non-target damage to winter grain crops
Source: PLoS One. 2025 Aug 21;20(8):e0330225. doi: 10.1371/journal.pone.0330225 (PMC12370053; doi:10.1371/journal.pone.0330225)
Supplement: S4 Table — (DOCX) [file pone.0330225.s005.docx]

| **Source** | **DF** | **SDWI** | **RDWI** | **SLI** | **RLI** |
| --- | --- | --- | --- | --- | --- |
| Block | 2 | 58 | 120 | 62 | 22 |
| Crop (C) | 5 | 1418 | 3573 | 903 | 1735 |
| Dose (D) | 7 | 39327 | 52682 | 39115 | 72877 |
| Herbicide (H) | 3 | 18773 | 6789 | 8795 | 11356 |
| C x D | 35 | 278 | 298 | 278 | 268 |
| C x H | 15 | 14197 | 11444 | 12080 | 7559 |
| D x H | 21 | 2422 | 1596 | 1492 | 2405 |
| C x D x H | 105 | 677 | 745 | 946 | 429 |
| all effects were highly significant (P < 0.001 by Tukey’s HSD test).  *Note-* DF denotes degrees of freedom, SDWI-Shoot dry weight inhibition, RDWI-Root dry weight inhibition, SLI-Shoot length inhibition, RLI-Root length inhibition. | | | | | |

**S4 Table.** Three factor analysis of variance results (mean squares and significance) for the effect of crop species, herbicides, herbicide doses and their interactions on crop growth responses.
