# Supplementary material for: Herbicide dose-response thresholds in sands to assess the risk of non-target damage to winter grain crops
Source: PLoS One. 2025 Aug 21;20(8):e0330225. doi: 10.1371/journal.pone.0330225 (PMC12370053; doi:10.1371/journal.pone.0330225)
Supplement: S5 Table — (DOCX) [file pone.0330225.s006.docx]

**S5 Table.** Two factor analysis of variance results (mean squares and significance) for the effect of crop species, herbicides and their interactions on crop growth responses at label rate.

| **Source** | **DF** | **SDWI** | **RDWI** | **SLI** | **RLI** |
| --- | --- | --- | --- | --- | --- |
| Block | 2 | 95 | 234 | 0.9 | 20 |
| Crop (C) | 5 | 1386*** | 942* | 344.4** | 1172*** |
| Herbicide (H) | 3 | 6136*** | 7626*** | 2839.8*** | 4858*** |
| C x H | 15 | 3400*** | 3529*** | 2920*** | 2211*** |
| all effects were highly significant (P < 0.001 by Tukey’s HSD test).  *Note-* DF denotes degrees of freedom, SDWI-Shoot dry weight inhibition, RDWI-Root dry weight inhibition, SLI-Shoot length inhibition, RLI-Root length inhibition.  The mean squares values derived from the ANOVA results represented the variance in crop growth responses associated with each factor (crop species, herbicides, herbicide doses) and their interactions, thereby offering insights into the significant of these factors. | | | | | |
