# Supplementary material for: Herbicide dose-response thresholds in sands to assess the risk of non-target damage to winter grain crops
Source: PLoS One. 2025 Aug 21;20(8):e0330225. doi: 10.1371/journal.pone.0330225 (PMC12370053; doi:10.1371/journal.pone.0330225)
Supplement: S6 Table — (DOCX) [file pone.0330225.s007.docx]

**S6 Table.** Estimated dose-response thresholds to clopyralid herbicide (µg kg^-1^soil) causing 20% (ED_20_) inhibition to shoot and root parameters of tested species at 4 weeks after sowing.

| **Crops** | **Shoot biomass** | **Root biomass** | **Shoot length** | **Root length** |
| --- | --- | --- | --- | --- |
|  | **ED_20_ and**  **95% CI** | **ED_20_ and**  **95% CI** | **ED_20_ and**  **95% CI** | **ED_20_ and**  **95% CI** |
| Canola | 6.5 (0.2-192.4) | 2.8 (0.01-1200) | 24067 (254-2283100) | 0.7 (0.002-239.3) |
| Chickpea | 1.7 (0.8-3.5) | 0.6 (0.2-2.2) | 2.9 (1.6-5.4) | 0.9 (0.5-1.9) |
| Fieldpea | 0.5 (0.2-1.2) | 8.5 (4.5-16.1) | 3.1 (1.2-8.3) | 2.2 (1.2-3.8) |
| Lentil | 1.5 (0.5-4.8) | 1.5 (0.2-15.3) | 7.6 (5.6-10.3) | 2.5 (0.6-10.5) |
| Lupin | 0.3 (0.1-0.9) | 0.003 (2.3E-06-3.9) | 7.9 (3.4-18.3) | 0.8 (0.4-1.9) |
| Wheat | 605.3 (219.8-1667.3) | 481.4 (315-647.8) | 586 (NaN) | 30.9 (10.9-87.8) |
