# Supplementary material for: Herbicide dose-response thresholds in sands to assess the risk of non-target damage to winter grain crops
Source: PLoS One. 2025 Aug 21;20(8):e0330225. doi: 10.1371/journal.pone.0330225 (PMC12370053; doi:10.1371/journal.pone.0330225)
Supplement: S7 Table — (DOCX) [file pone.0330225.s008.docx]

**S7 Table.** Estimated dose-response thresholds to pyroxasulfone herbicide (µg kg^-1^soil) causing 20% (ED_20_) inhibition to shoot and root parameters of tested species at 4 weeks after sowing.

| **Crops** | **Shoot biomass** | **Root biomass** | **Shoot length** | **Root length** |
| --- | --- | --- | --- | --- |
|  | **ED_20_ and**  **95% CI** | **ED_20_ and**  **95% CI** | **ED_20_ and**  **95% CI** | **ED_20_ and**  **95% CI** |
| Canola | 1.9 (0.9-3.9) | 2.2 (1.2-4.0) | 7.8 (5.1-12.1) | 1.0 (0.4-2.2) |
| Chickpea | 930.2 (NaN) | 50.1 (6.4-389.9) | 1095.6 (184-6524.7) | 8.4 (5.2-13.5) |
| Fieldpea | 1865 (339-10278) | 47.5 (8.3-272.0) | 530.4 (305.1-921.8) | 6.0 (3.3-10.8) |
| Lentil | 263.4 (68.5-1013.5) | 1.6 (0.7-4.1) | 110.7 (73-168) | 1.9 (0.9-4) |
| Lupin | 7.2 (1.8-28.6) | 6.0 (1.2-29.5) | 63.7 (33.5-121.1) | 2.2 (1-5.2) |
| Wheat | 18.9 (2.6-137.4) | 4.5 (1.0-20.2) | 212.3 (147.6-305.3) | 3.5 (2.3-5.3) |
