# Supplementary material for: Herbicide dose-response thresholds in sands to assess the risk of non-target damage to winter grain crops
Source: PLoS One. 2025 Aug 21;20(8):e0330225. doi: 10.1371/journal.pone.0330225 (PMC12370053; doi:10.1371/journal.pone.0330225)
Supplement: S8 Table — (DOCX) [file pone.0330225.s009.docx]

**S8 Table.** Estimated dose-response thresholds to propyzamide herbicide (µg kg^-1^soil) causing 20% (ED_20_) inhibition to shoot and root parameters of tested species at 4 weeks after sowing.

| **Crops** | **Shoot biomass** | **Root biomass** | **Shoot length** | **Root length** |
| --- | --- | --- | --- | --- |
|  | **ED_20_ and**  **95% CI** | **ED_20_ and**  **95% CI** | **ED_20_ and**  **95% CI** | **ED_20_ and**  **95% CI** |
| Canola | 194 (134.1-281.6) | 393.2 (227-681) | 82 (12.2-545.7) | 213 (155.8-292) |
| Chickpea | 2446.8  (1973.5-3033.7) | 803.9 (566.4-1141) | 2367.5  (1825.6-3070.1) | 324.1  (250.9-418.7) |
| Fieldpea | 1571.5  (1205.8-2048.2) | 1157.1 (88.3-1945) | 2049.4  (1559.8-2692.8) | 208.0  (117.2-369.3) |
| Lentil | 1271.6  (909.7-1777.4) | 982.1 (783.4-1231.2) | 1976.7  (1716.1-2276.9) | 975.2  (833.7-1140.7) |
| Lupin | 247.7 (116-527) | 393.2 (227-681) | 621.8 (524-738) | 172.3 (125-237) |
| Wheat | 46.3 (40.5-53) | 26.9 (20-36) | 51.1 (44.6-58.5) | 12.6 (7.5-21.2) |
