# Supplementary material for: Herbicide dose-response thresholds in sands to assess the risk of non-target damage to winter grain crops
Source: PLoS One. 2025 Aug 21;20(8):e0330225. doi: 10.1371/journal.pone.0330225 (PMC12370053; doi:10.1371/journal.pone.0330225)
Supplement: S9 Table — (DOCX) [file pone.0330225.s010.docx]

**S9 Table.** Estimated dose-response thresholds to trifluralin herbicide (µg kg^-1^soil) causing 20% (ED_20_) inhibition to shoot and root parameters of tested species at 4 weeks after sowing.

| **Crops** | **Shoot biomass** | **Root biomass** | **Shoot length** | **Root length** |
| --- | --- | --- | --- | --- |
|  | **ED_20_ and**  **95% CI** | **ED_20_ and**  **95% CI** | **ED_20_ and**  **95% CI** | **ED_20_ and**  **95% CI** |
| Canola | 380.1  (272.6-529.8) | 523.2  (355.6-769.7) | 331.1  (180.5-607.2) | 437.4  (323.4-591.6) |
| Chickpea | 550.5  (317.6-954.2) | 287.7  (159.5-518.9) | 1042.2  (733.5-1480.9) | 329.1  (264.5-409.5) |
| Fieldpea | 259.1  (200.5-334.9) | 148.5  (88.4-249.7) | 416.9  (303.3-573.1) | 328.9  (284.8-379.9) |
| Lentil | 215.8  (108.7-428.4) | 103.9  (28-385.9) | 694.9  (606.5-796.2) | 115.1  (48.3-274.5) |
| Lupin | 481.6  (203.9-1137.4) | 744.1  (143.8-3850) | 2014.0  (1445.1-2806.6) | 241.8  (174.8-334.7) |
| Wheat | 371.2  (326.7-421.9) | 184.2  (107.5-315.5) | 386.0  (349.7-426.1) | 129.1  (106.3-156.7) |
